# Identification of key serum biomarkers for the diagnosis and metastatic prediction of osteosarcoma by analysis of immune cell infiltration

**DOI:** 10.1186/s12935-022-02500-6

**Published:** 2022-02-12

**Authors:** Zhihao Chen, Liubing Li, Ziyuan Li, Xi Wang, Mingxiao Han, Zongshuai Gao, Min Wang, Gangfeng Hu, Xiaolu Xie, Hong Du, Zonggang Xie, Haifang Zhang

**Affiliations:** 1 Department of Orthopedics, The Second Affiliated Hospital of Soochow University, Suzhou, China; 2 Department of Clinical Laboratory, The Second Affiliated Hospital of Soochow University, Suzhou, China; 3 Department of Transfusion Medicine, Shanghai Jiao Tong University Affiliated Sixth People’s Hospital, Shanghai, China

**Keywords:** circRNA, miRNA, Immunotherapy, Biomarkers, Osteosarcoma

## Abstract

**Background:**

The role of circular RNAs (circRNAs) and microRNAs (miRNAs) in osteosarcoma (OS) development has not been fully elucidated. Further, the contribution of the immune response to OS progression is not well defined. However, it is known that circRNAs and miRNAs can serve as biomarkers for the diagnosis, prognosis, and therapy of many cancers. Thus, the aim of this study was to identify novel key serum biomarkers for the diagnosis and metastatic prediction of OS by analysis of immune cell infiltration and associated RNA molecules.

**Methods:**

Human OS differentially expressed circRNAs (DEcircRNAs), differentially expressed miRNAs (DEmiRNAs), and differentially expressed mRNAs (DEmRNAs) were identified by analysis of microarray data downloaded from Gene Expression Omnibus (GEO) datasets. Further, characteristic patterns of OS-infiltrating immune cells were analyzed. On this basis, we identified statistically significant transcription factors. Moreover we performed pathway enrichment analysis, constructed protein–protein interaction networks, and devised competitive endogenous RNA (ceRNA) networks. Biological targets of the ceRNA networks were evaluated and potential OS biomarkers confirmed by RT-qPCR analysis of the patients’ serum.

**Results:**

Seven differentially expressed circRNAs, 166 differentially expressed miRNAs, and 175 differentially expressed mRNAs were identified. An evaluation of cellular OS infiltration identified the highest level of infiltration by M0 macrophages, M2 macrophages, and CD8+ T cells, with M0 macrophages and CD8+ T cells as the most prominent. Significant patterns of tumor-infiltrating immune cells were identified by principal component analysis. Moreover, 185 statistically significant transcription factors were associated with OS. Further, in association with immune cell infiltration, hsa-circ-0010220, hsa-miR-326, hsa-miR-338-3p, and FAM98A were identified as potential novel biomarkers for OS diagnosis. Of these, FAM98A had the most promise as a diagnostic marker for OS and OS metastasis. Most importantly, a novel diagnostic model consisting of these four biomarkers (hsa-circ-0010220, hsa-miR-326, hsa-miR-338-3p, and FAM98A) was established with a 0.928 AUC value.

**Conclusions:**

In summary, potential serum biomarkers for OS diagnosis and metastatic prediction were identified based on an analysis of immune cell infiltration. A novel diagnostic model consisting of these four promising serum biomarkers was established. Taken together, the results of this study provide a new perspective by which to understand immunotherapy of OS.

**Supplementary Information:**

The online version contains supplementary material available at 10.1186/s12935-022-02500-6.

## Introduction

Osteosarcoma (OS) is the most common primary malignant tumor of bone in young people [1]. Surgery, chemotherapy, and radiotherapy have significantly improved the survival rate of patients with OS [2]. Despite these improvements, the prognosis for patients with bone tumors remains very poor [3]. Most patients with OS will eventually die from metastasis [4]. At present, alkaline phosphatase (ALP) and lactate dehydrogenase (LDH) are the most widely used serum biomarkers for OS diagnosis, even though they have unsatisfactory sensitivity and specificity [5–7]. Therefore, it is extremely important to find promising biomarkers for early-stage OS diagnosis, making it possible to predict metastasis and disease progression.

Circular RNA (circRNA) is a newly discovered non-coding form of RNA with a covalent closed loop structure [8], without a 5′ cap structure or a 3′ poly(A) tail. CircRNA is therefore resistant to exonucleases and consequently an ideal biomarker [9]. The competitive endogenous RNA (ceRNA) network is considered the primary mechanism by which circRNA performs biological functions [10]. For example, Pan et al*.* found that circ_0028171 acts as a sponge for microRNA (miR)-218-5p, increasing the expression of IKBKB and promoting the progression of OS. MiR-218-5p has been described as a novel biomarker for early diagnosis of OS [11]. Li et al*.* reported that hsa_circ_0000073 can act as a sponge to inhibit miR-145-5p- and miR-151-3p-mediated down-regulation of NRAS, which promotes proliferation, migration, invasion, and methotrexate (MTX) resistance of OS cells [12]. Zhang et al*.* found that high expression of hsa_circ_0136666 predicted a poor prognosis and promoted the development of OS through the miR-593-3p/ZEB2 pathway [13]. Although circRNA plays a potentially important role in OS, the function and complex mechanisms of action for most circRNAs have not been fully elucidated.

The tumor microenvironment (TME) provides nutrients and growth factors for the proliferation and metastasis of tumor cells, and limits the early detection of tumors and the efficacy of immunotherapy [14]. The OS microenvironment is complex, with the presence of various cells, growth factors, and cytokines [15]. CIBERSORT is a new biological information tool. Through a deconvolution algorithm developed by Bindea et al*.*, CIBERSORT can estimate the cell composition of complex tissues based on standardized gene expression data [16, 17]. This method has been validated by flow cytometry for breast and lung cancer and can be used to analyze large-scale gene expression profile data [18–20]. Because the microenvironment plays an important role in tumor development, analysis of the OS immune microenvironment may provide for a better understanding of the pathogenesis of OS. Further, immunotherapy has become an important treatment option for OS [21–23]. Most importantly, circRNA has become a promising cancer immunotherapy biomarker, especially for the TME [24, 25]. Investigations of ceRNA networks within the OS TME are limited, with few reports.

In this study, we collected in vivo circRNA, miRNA, and mRNA expression profiles of OS patients and healthy individuals from the Gene Expression Omnibus (GEO) dataset. Significant characteristic patterns of tumor-infiltrating immune cells and their functional potential were identified. Based on these results, we constructed protein–protein interaction (PPI) and competitive endogenous RNA (ceRNA) networks. We verified the targets of the ceRNA network by RT-qPCR of patients’ serum and found that hsa-circ-0010220, hsa-miR-326, hsa-miR-338-3p, and FAM98A of the ceRNA network were associated with immune cell infiltration. Of these circRNAs, FAM98A had the most potential for diagnosis and metastatic OS prediction. Most importantly, a promising diagnostic model consisting of these four targets (hsa-circ-0010220, hsa-miR-326, hsa-miR-338-3p, and FAM98A) was identified with a 0.928 area under the curve (AUC) value.

## Materials and methods

### Raw data

The raw datasets—GSE140256, GSE65071, GSE16088, GSE21257, GSE33382, and GSE124768—were obtained from the Gene Expression Omnibus (GEO; https://www.ncbi.nlm.nih.gov/geo/), which is an online public gene data repository for high-throughput sequencing research. GSE140256, a circRNA expression profile, includes three cancer tissue samples and three para-cancer samples. MiRNA sequencing data obtained from GSE65071 included 20 serum samples from patients with OS and 15 normal serum samples. GSE16088, containing mRNA expression profile data, included 17 cancer tissue samples and six normal tissue samples. Together, GSE21257, GSE33382, and GSE124768 included 56 non-metastatic patients and 71 metastatic patients or patients metastatic within five years. These datasets were used to explore differences between non-metastatic and metastatic patients. This study did not require any ethical review or informed consent because of the public availability of GEO data.

### Identification of DEcircRNAs, DEmiRNAs, and DEmRNAs

Differentially expressed circRNAs (DEcircRNAs), differentially expressed miRNAs (DEmiRNAs), and differentially expressed mRNAs (DEmRNAs) were identified by differences in expression between normal and OS samples within the microarray data. The *P*-value and the absolute log value of fold-change (log|FC|) were analyzed in R language with the limma package. A log|FC| > 1.0 and *P* < 0.05 were used to define differentially expressed genes (DEGs).

### Tumor-infiltrating immune cell analysis

We used Cibersort software to analyze DEmRNAs differentially expressed in OS and normal tissues. The distribution characteristics of 22 immune cells were calculated by the deconvolution method of Cibersort software. The 22 immune cell types included activated dendritic cells (DCs), resting DCs, activated mast cells, resting mast cells, activated natural killer cells (NKs), resting NKs, activated memory CD4+ T cells, resting CD4+ T cells, naïve CD4+ T cells, regulatory T cells (Tregs), T follicular helper cells (Tfhs), gamma delta T cells (Tgds), CD8+ T cells, eosinophils, neutrophils, monocytes, macrophages (M0s), type 1 macrophages (M1), type 2 macrophages (M2), memory B cells, naïve B cells, and plasma cells. Values of *P* < 0.05 were considered statistically significant.

### Analysis of differential expressed transcription factors

Based on the identified DEmiRNAs, FunRich (Version 3.1.3) was used to analyze and visualize the differentially expressed transcription factors. FunRich is a stand-alone software tool used mainly for functional enrichment and interaction network analysis of genes and proteins.

### Pathway enrichment analysis

We used GO annotation (http://www.geneontology.org) and KEGG pathway analysis to determine the potential function of the DEmRNAs. *P*-values < 0.05, as a screening condition, were considered highly credible using R language and the clusterProfiler package.

### Construction of the ceRNA networks

By differential analysis of the microarray data, DEcircRNAs were predicted by the tissue-specific circRNA database (TSCD) (http://gb.whu.edu.cn/TSCD). DEmiRNA target genes were predicted by miRDB, miRTarBase, and TargetScan databases. Downstream molecules predicted by all three databases were identified as target genes of DEcircRNAs and DEmiRNAs.

We used the DEcircRNAs and DEmiRNAs predicted target genes to intersect with identified differentially expressed downstream genes to further screen prediction results. Finally, we used these prediction results to construct ceRNA interaction networks, which were visualized with Cytoscape (Version 3.8.0).

### PPI network and clustered sub-network construction

Protein interactions provide a means by which to understand underlying pathological mechanism of OS. In this study, we used the Search Tool for the Retrieval of Interacting Genes/Proteins (STRING) database (https://string-db.org/) to construct a PPI network. In this manner, clustered subnetworks and hub genes were identified using Molecular Complex Detection (MCODE) and CytoHubba in Cytoscape (Version 3.8.0).

### Verification of diagnostic specificity using pan-cancer analysis

The function of non-coding (nc)RNA is the regulation of target genes. Exploration of these target genes provides valuable information about their contribution to pathology. Expression data for target genes of the ceRNA networks were obtained as Fragments per Kilobase of transcript per Million mapped reads (FPKM) from the Cancer Genome Atlas (TCGA) and the Genotype-Tissue Expression (GTEx) database. We analyzed 32 different cancer types and 10,967 samples from TCGA and 17,382 samples from GTEx, including kidney renal clear cell carcinoma (KIRC); kidney renal papillary cell carcinoma (KIRP); kidney chromophobe (KICH); brain lower grade glioma (LGG); glioblastoma multiforme (GBM); breast cancer (BRCA); lung squamous cell carcinoma (LUSC); lung adenocarcinoma (LUAD); rectum adenocarcinoma (READ); colon adenocarcinoma (COAD); uterine carcinosarcoma (UCS); uterine corpus endometrial carcinoma (UCEC); ovarian serous cystadenocarcinoma (OV); head and neck squamous carcinoma (HNSC); thyroid carcinoma (THCA); prostate adenocarcinoma (PRAD); stomach adenocarcinoma (STAD); skin cutaneous melanoma (SKCM); bladder urothelial carcinoma (BLCA); liver hepatocellular carcinoma (LIHC); cervical squamous cell carcinoma and endocervical adenocarcinoma (CESC); adrenocortical carcinoma (ACC); pheochromocytoma and paraganglioma (PCPG); sarcoma (SARC); pancreatic adenocarcinoma (PAAD); esophageal carcinoma (ESCA); testicular germ cell tumors (TGCT); thymoma(THYM); uveal melanoma (UVM); lymphoid neoplasm diffuse large b-cell lymphoma (DLBC); cholangiocarcinoma (CHOL). The diagnostic specificity of gene targets we provided was better hightlight between different types of cancer.

### Verification of metastatic characteristics

Metastasis has always been an important obstacle to OS treatment. There are no effective biomarkers by which to diagnose early-stage OS, nor are biomarkers available for prediction of OS pathological progression. The patient clinical data from GEO datasets (GSE21257, GSE33382, and GSE124768) were employed to analyze the relationships among the identified potential targets in OS and metastasis using the limma package of R language. The importance of these gene targets was evaluated by comparison of non-metastatic patients with metastatic patients and with patients who had metastases within 5 years. Fold-change > 1.5 and a *P*-value < 0.05 were used as selection criteria.

### Patients’ serum diagnostic verification

Specific validation primers (Additional file 1: Table S1) for several differentially expressed RNAs (DERNAs) were designed based on linear transcript sequence. Serum samples for 19 OS patients and 19 healthy individuals were obtained. Ethical approval was obtained from the ethics committee of the Second Affiliated Hospital of Soochow University. Total RNA was extracted from the tissue samples using TRIzol reagentTRIzol (Invitrogen USA) and treated with RNase-free DNase I (Vazyme Biotech Co., China) to eliminate traces of DNA. Real-time PCR was performed with an Applied Biosystems StepOnePlus Real-Time PCR System (Thermo Fisher Scientific, USA) using qPCR SYBR Green master mix (Vazyme Biotech Co., China). The expression levels of DEcircRNAs and DEmRNAs were normalized to the endogenous control, human glyceraldehyde-3-phosphate dehydrogenase (GAPDH). The expression levels of DEmiRNAs were normalized to the endogenous control of U6. The receiver operating characteristic (ROC) curve and logistic regression package in SPSS were used to construct the diagnostic model.

### Statistical analysis

Bioinformatic analysis was performed using R software (version 4.0.1), FunRich (version 3.1.3), and Cytoscape (version 3.8.0). GraphPad Prism (GraphPad Software, USA) and SPSS (IBM, USA) were used for statistical analysis of qRT-PCR. Quantitative data were analyzed for statistical significance by t-test and expressed as the mean ± SD. A *P*-value < 0.05 was accepted as statistically significant.

## Results

### Identification of DEcircRNAs, DEmiRNAs, and DEmRNAs

**Fig. 1:**
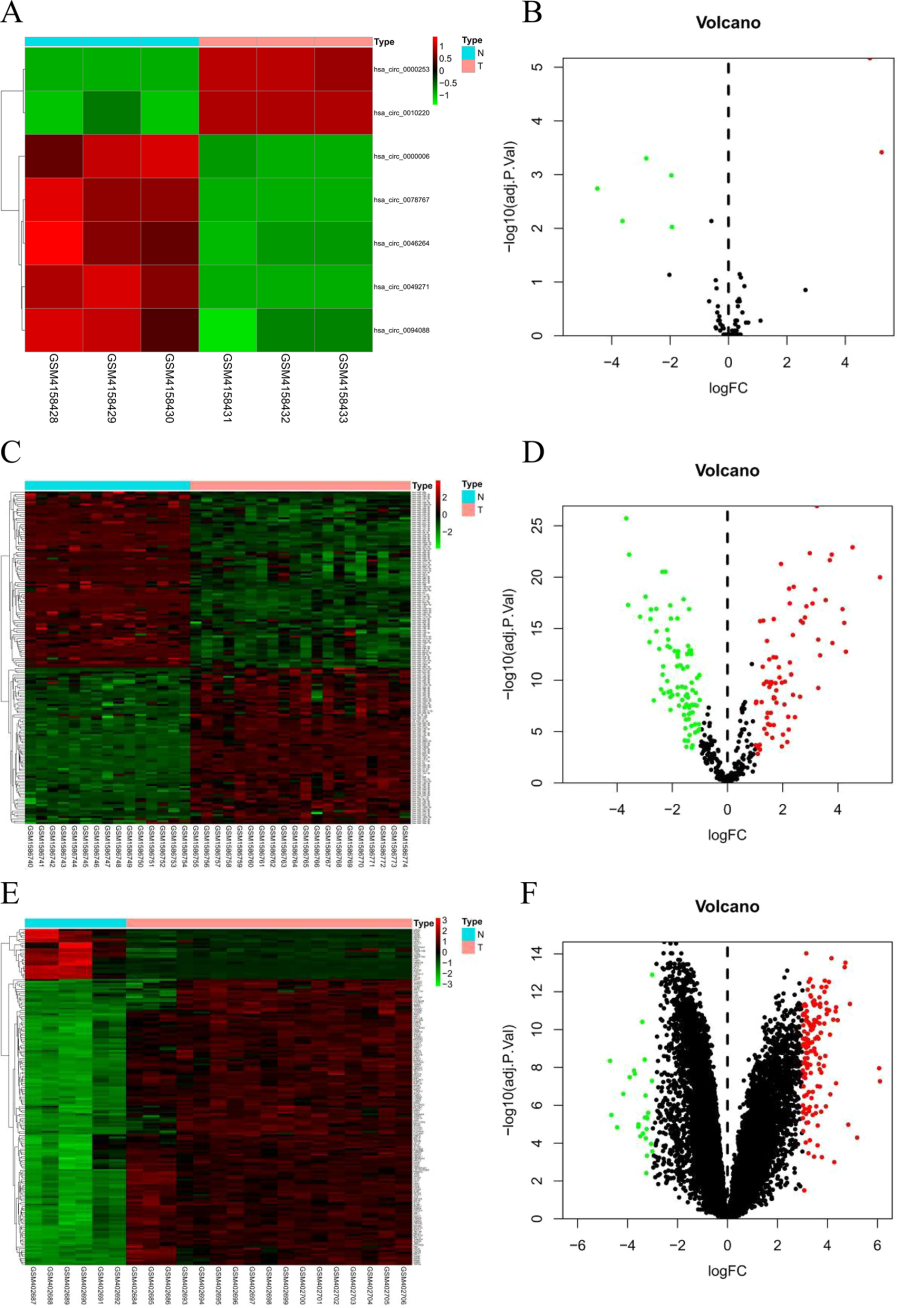
Characteristics of differentially-expressed genes(DEGs). **A** Unsupervised clustering analysis of differentially-expressed (DE) circular RNAs (circRNAs). Red dots indicate significantly up-regulated circRNAs, green dots indicate significantly down-regulated circRNAs. **B** Volcano plots of circRNAs. Red dots indicate up-regulated DEcircRNAs, green dots indicate down-regulated DEcircRNAs, black dots indicate non-differentially expressed circRNAs. **C** Unsupervised clustering analysis of DE micro RNAs (miRNAs). Red dots indicate significantly up-regulated miRNAs, green dots indicate significantly down-regulated miRNAs. **D** Volcano plots of miRNAs. Red dots indicate up-regulated DEmiRNAs, green dots indicate down-regulated DEmiRNAs, black dots indicate non-differentially expressed miRNAs. **E** Unsupervised clustering analysis of the DE messenger RNAs (mRNAs). Red dots indicate significantly up-regulated mRNAs, green dots indicate significantly down-regulated mRNAs. **F** Volcano plots of mRNAs. Red dots indicate up-regulated DEmRNAs, green dots indicate down-regulated DEmRNAs, black dots indicate non-differentially expressed mRNAs

We performed a comprehensive bioinformatics analysis to identify key OS biomarkers (flow chart is shown in Additional file 2: Fig. S1). Values of log|FC| > 1.0 and *P* < 0.05 were the selection criteria for DEGs. In the GSE140256 dataset, four up-regulated circRNAs and three down-regulated circRNAs were identified, based on the comprised three cancer tissue samples and three para-cancer samples (Fig. 1A, B). The GSE65071 dataset, consisting of 20 serum samples from patients with OS and 15 normal serum samples, had 78 up-regulated miRNAs and 88 down-regulated miRNAs (Fig. 1C, D). The mRNA expression profile data were derived from 17 cancer tissue samples and six normal samples. The GSE16088 dataset identified 175 DEmRNAs of which 149 mRNAs were up-regulated and 26 mRNAs were down-regulated (Fig. 1E, F).

### Tumor-infiltrating immune cell analysis

**Fig. 2:**
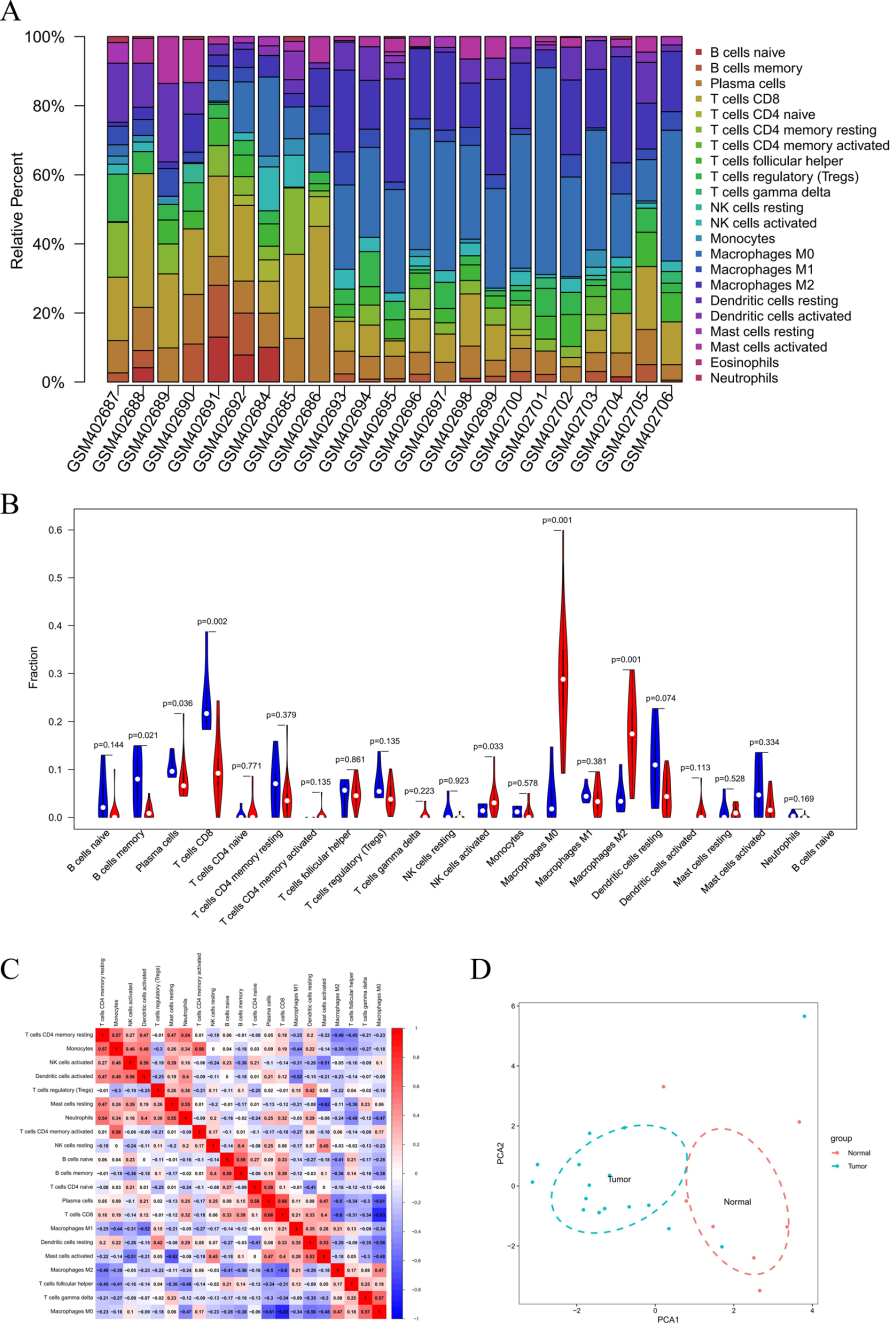
Tumor-infiltrating immune cells analyzed by the identified DEmRNAs. **A** The mean proportion of 22 immune cells in the 17 OS patient tissues and six normal human tissues. **B** Violin plot of OS patient tissues (red) and normal human tissues (blue). The P values showed different infiltrating types of immune cells. **C** Correlation matrix of 21 immune cell proportions and immune/stromal score. Variables have been ordered by average linkage clustering. **D** Principal component analysis of immune cell infiltration patterns of 22 types of immune cell between the OS patient tissues and normal human tissues. Red circles indicate normal human tissues, blue circles indicate OS patient tissues

We used Cibersort to evaluate the distribution of immune cells based on the data derived from 17 OS patient tissues and six normal human tissues. The immune cell types with the greatest degree of OS patient infiltration were M0 macrophages, M2 macrophages, and CD8+ T cells (Fig. 2A). Compared to normal tissues, M0 macrophages (*P* = 0.010), M2 macrophages (*P* = 0.010), CD8+ T cells (*P* = 0.020), memory B cells (*P* = 0.021), plasma cells (*P* = 0.036), and activated NK cells (*P* = 0.033) differed in OS patient tissues (*P* < 0.05) (Fig. 2B). In the tissues of OS patients, immune cells with a larger positive correlation coefficient included CD8+ T cells and plasma cells (0.66), memory B cells and naïve B cells (0.59), as well as plasma cells and naïve CD4+ T cells (0.58). Immune cells with larger negative correlation coefficients included M0 macrophages and CD8+ T cells (0.83), resting mast cells and activated mast cells (0.62), as well as plasma cells and M0 macrophages (0.61) (Fig. 2C). Most importantly, results of principal component analysis (PCA) using the linear dimensionality reduction method for processing of multivariate data, showed the infiltration pattern of 22 types of immune cell to effectively distinguish OS patients from healthy controls (Fig. 2D).

### Analysis of differentially expressed transcription factors

**Fig. 3:**
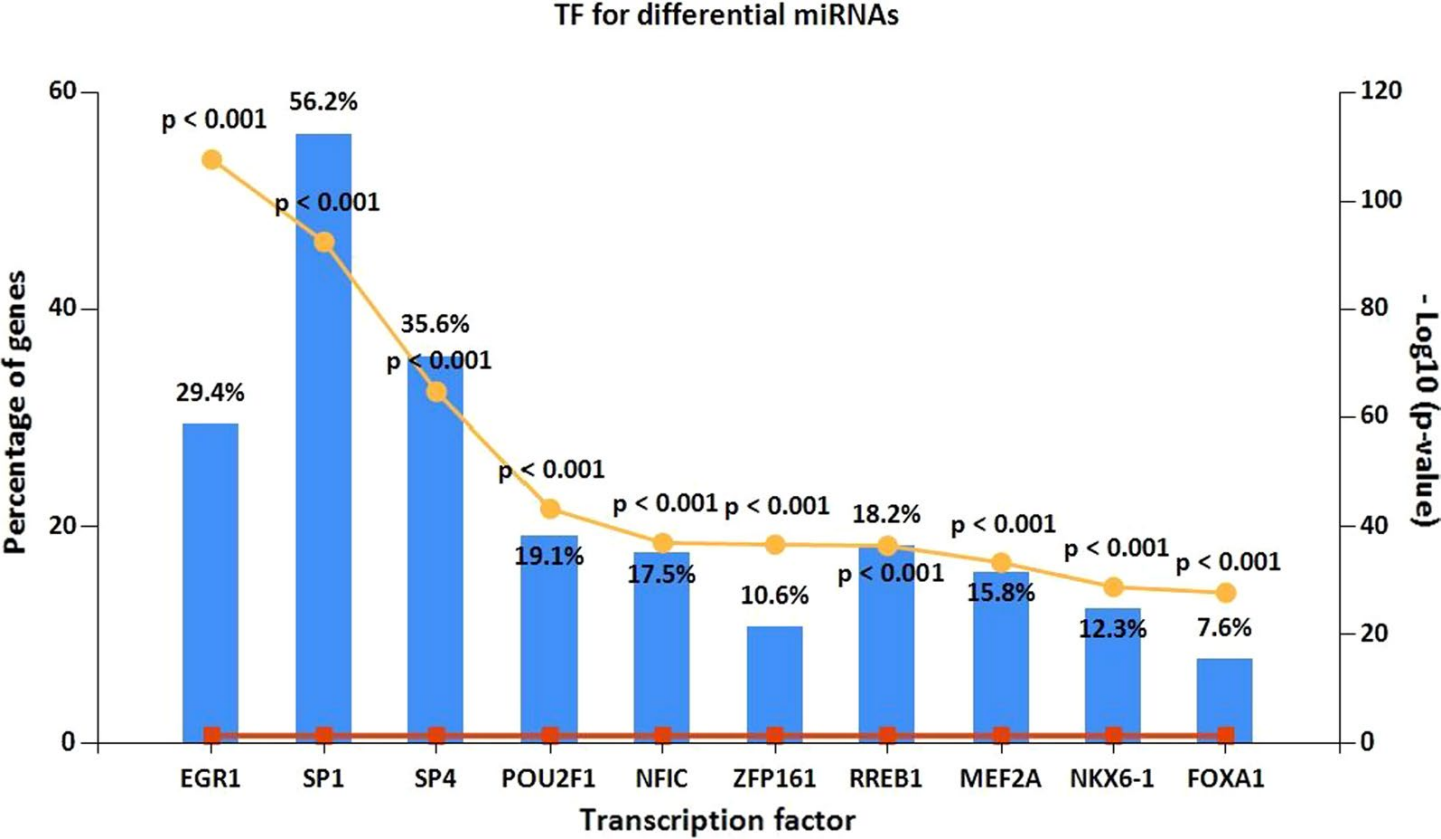
Analysis of differentially-expressed transcription factors associated with the identified DEmiRNAs. EGR1, SP1, SP4, POU2F1 and NFIC are the most significant molecules in OS

To assess potential tumor immune biomarkers, transcription factor analysis was performed. Transcription factors, as direct downstream targets of miRNA, are essential molecular regulators of the cancer immune response. We analyzed differentially expressed transcription factors associated with the 166 identified DEmiRNAs, and found 185 statistically significant transcription factors. Among these, EGR1, SP1, SP4, POU2F1, and NFIC were the most significant molecules related to OS. EGR1 is the most significant target among the transcription factors we identified, with SP1 found to be the largest percentage (Fig. 3). Because of the importance of transcription factors, their potential in the OS immune response deserves further exploration.

### Pathway enrichment analysis

**Fig. 4:**
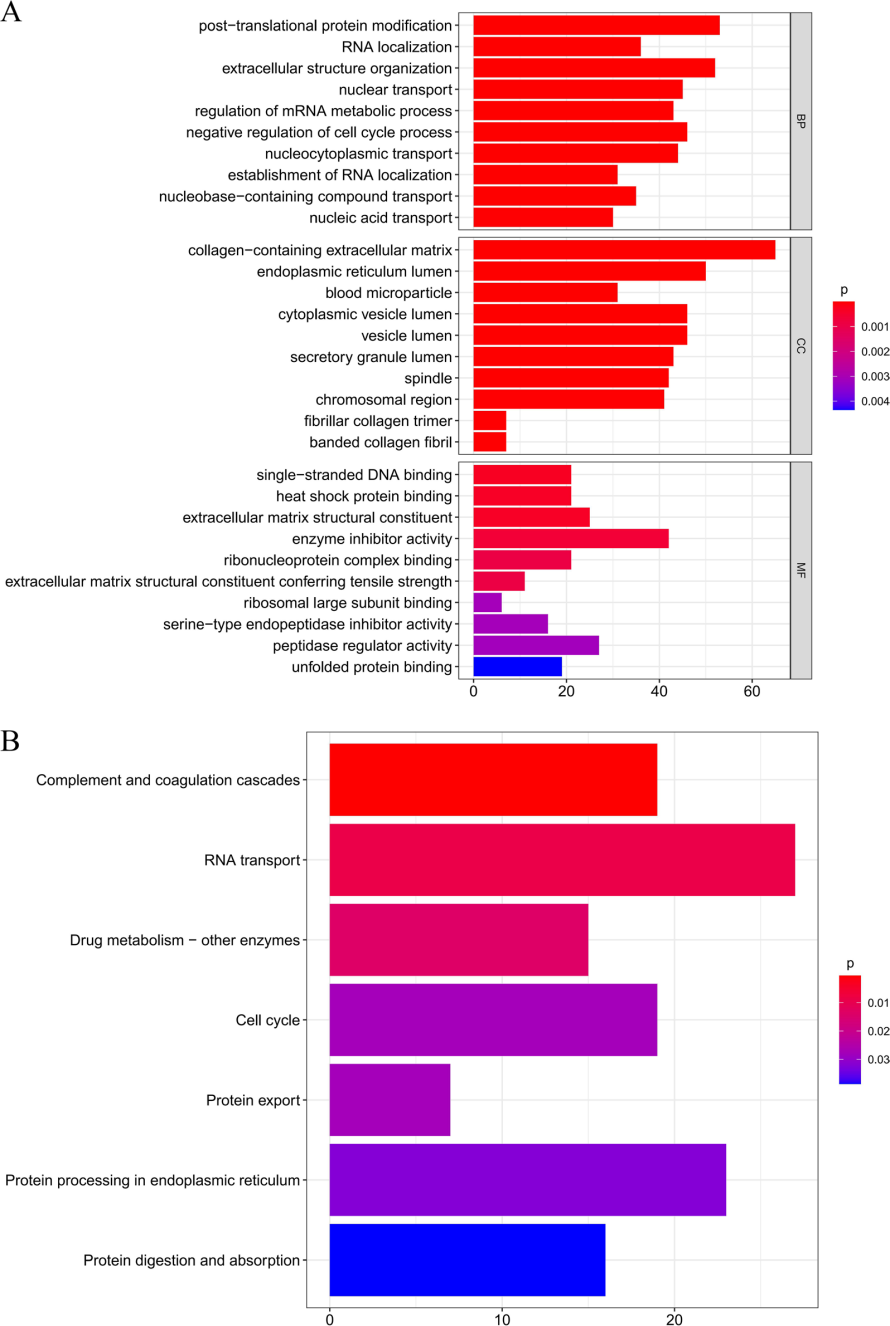
Pathway enrichment analysis. **A** GO enrichment analysis of the DEmRNAs in biological process (BP), cellular component (CC) and molecular function (MF) subgroups. **B** KEGG enrichment analysis of DEmRNAs

To better understand the potential biological function of the OS identified DEmRNAs, GO annotation and KEGG pathway analysis were performed. Through GO annotation analysis, we found that DEmRNAs were significantly enriched in post-translational protein modification, RNA localization, and extracellular structure organization within the biological process (BP) subgroup. Collagen-containing extracellular matrix, endoplasmic reticulum lumen, and blood micro-particle were the most significant GO terms in the cellular component (CC) subgroup. The top three GO processes were single-stranded DNA binding, heat shock protein binding, and extracellular matrix structural constituent in the molecular function (MF) subgroup for DEmRNAs (Fig. 4A).

For KEGG pathway enrichment analysis, complement and coagulation cascades, RNA transport, and drug metabolism, other enzymes were the most significant pathways enriched for DEmRNAs in OS (Fig. 4B).

### Construction of the ceRNA network

**Fig. 5:**
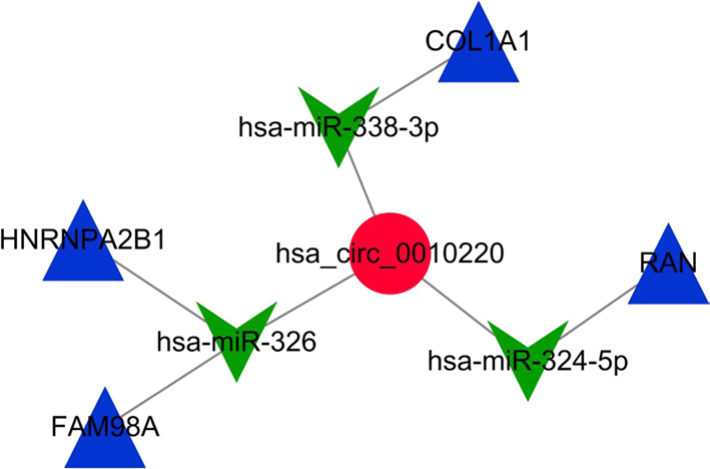
The interaction network of DEGs in OS. Based on the predicted results of circRNA–miRNA and miRNA–mRNA pairs, we constructed a ceRNA network including one circRNAs, three miRNAs and four mRNAs

To better understand OS endogenous regulation of DEGs, we predicted the downstream target genes of DEcircRNAs using the TSCD databases. At the circRNA level, miRNAs were predicted to be downstream of DEcircRNAs. We used the predicted miRNAs as candidate miRNAs. Candidate miRNAs were cross-compared with DEmiRNAs identified by microarray, with three intersecting miRNAs detected. At the miRNA level, 153 miRNAs were predicted of a possible 7580 mRNAs. After comparison, four intersecting mRNAs were identified. Based on the predicted results for circRNA–miRNA and miRNA–mRNA pairs, we constructed a final ceRNA network, which comprised one circRNA, three miRNAs, and four mRNAs (Fig. 5).

### Construction of the PPI network

**Fig. 6:**
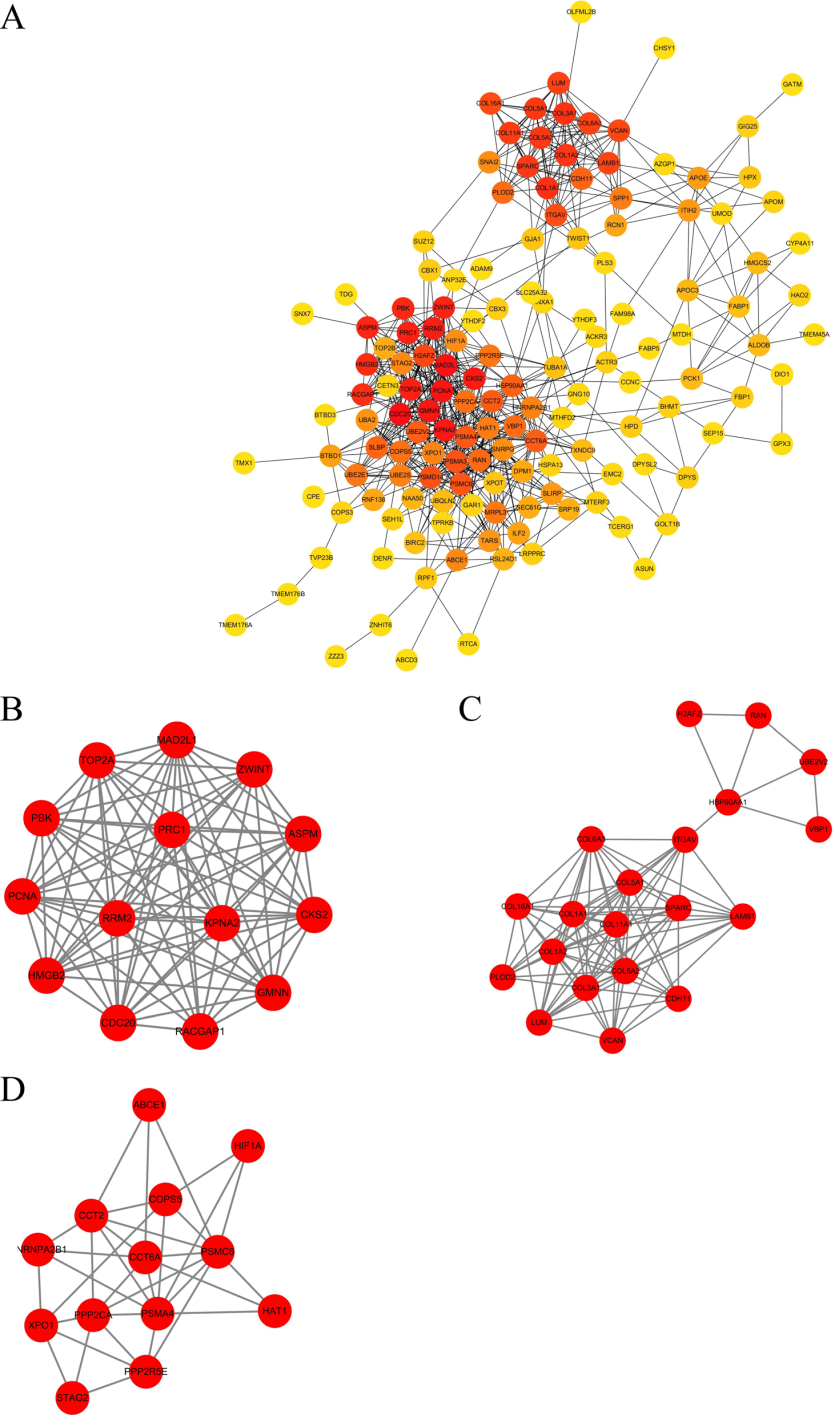
The PPI network of the target genes of the identified DEmRNAs. **A** The target genes of the identified DEmRNAs were ranked in the PPI network. The depth of red indicates the importance of genes in the network. **B** The most closely-clustered subnetwork was composed of 14 nodes and 90 edges. **C** The clustered subnetwork identified by the MCODE plug-in in Cytoscape had 20 nodes and 97 edges. **D** The clustered subnetwork identified by the MCODE plug-in in Cytoscape which had 13 nodes and 35 edges

We used the identified DEmRNAs to construct a PPI network. This network included 146 nodes and 687 edges, with the conditions that the comprehensive Gt score > 0.4 and unconnected points were removed. Among the 146 genes, 67 genes had a score > 100 when analyzed by the Maximal Clique Centrality (MCC) method in CytoHubba. The top five hub genes were CDC20, MAD2L1, PCNA, KPNA2, and PRB1 (Fig. 6A). In this study, we also defined the most closely clustered subnetwork by employing the MCODE plug-in of Cytoscape. The most closely clustered subnetwork was found to consist of 14 nodes and 90 edges (Fig. 6B). In addition, we also identified two other clustered subnetworks, of which one subnetwork contained 20 nodes and 97 edges and the other subnetwork 13 nodes and 35 edges (Fig. 6C, D). All of which were composed of up-regulated mRNAs.

### Pan-cancer analysis

**Fig. 7:**
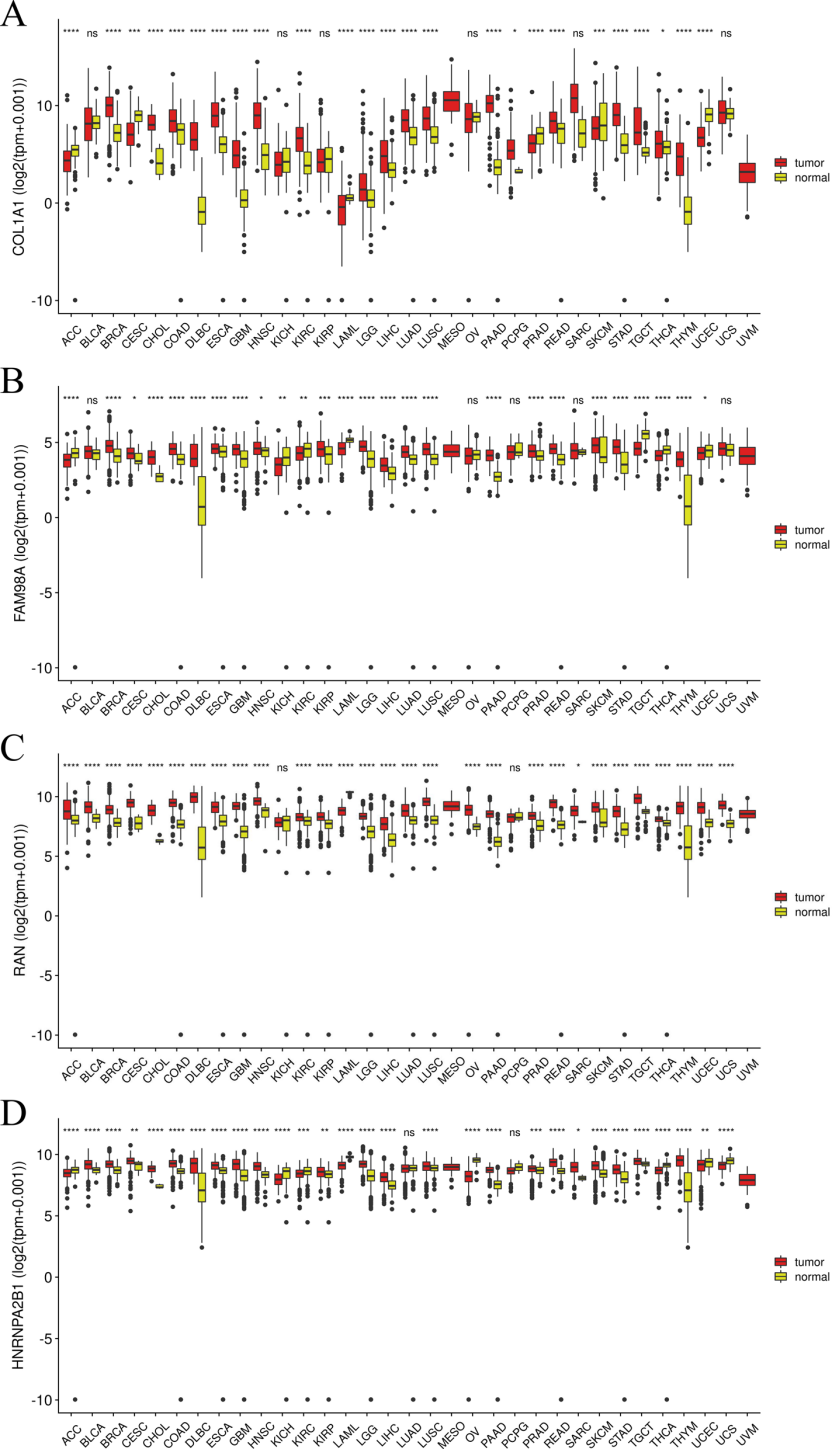
Pan-cancer analysis. **A** The expression level of COL1A1 in 32 different cancer types. **B** The expression level of FAM98A in 32 different cancer types. **C** The expression level of RAN in 32 different cancer types. **D** The expression level of HNRPHA2B1 in 32 different cancer types

Pan-cancer analysis was used to assess the diagnostic potential for target genes. Pan-cancer analysis demonstrated COL1A1 and FAM98A to have good specificity. There were no differences in the expression of COL1A1 in BLCA, KICH, KIRP, OV, SARC, or UCS. It is worth mentioning that COL1A1 did not show differential expression in SARC, which indicates that COL1A1 has better OS diagnostic specificity (Fig. 7A). FAM98A was not differentially expressed in BLCA, OV, PCPG, SARC, or UCS. In particular, the non-differential expression of FAM98A in SARC demonstrated its specificity for OS diagnosis (Fig. 7B). RAN and HNRNPA2B1 showed differential expression in most cancers. There were no significant expression differences for KICH and PCPG, or LUCAD and PCPG (Fig. 7C, D).

### Verification of metastatic characteristics

To verify the biological function of the target genes, public data from different world-side regions including Europe and America were employed to analyze the metastasis characteristics of the target genes. Metastatic patients and patients with tumors that metastasized within 5 years were compared to non-metastatic patients.

Two different GEO datasets (GSE21257 and GSE124768) demonstrated statistically significant differential expression of FAM98A between OS non-metastatic and metastatic patients. Two datasets (GSE21257 and GSE33382) were used to evaluate patients with potential metastasis. We found that FAM98A was differentially expressed by non-metastatic and patients with tumor metastasis within 5 years. Another target gene with good specificity, COL1A1, was not found to be different between non-metastatic and metastatic patients, or between non-metastatic and patients with tumor metastasis within 5 years (Table 1).

**Table 1:** Verification of metastasis characteristics

	GSE21257 non-metastasis vs metastasis		GSE124768 non-metastasis vs metastasis		GSE21257 non-metastasis vs metastasis within 5 years		GSE33382 non-metastasis vs metastasis within 5 years	
	logFC	P value	logFC	P value	logFC	P value	logFC	P value
COL1A1	0.176	0.354	0.398	0.186	0.233	0.174	0.261	0.144
FAM98A	1.330	0.003	0.936	0.009	0.817	0.037	1.020	0.005
RAN	− 0.130	0.422	0.512	0.024	− 0.145	0.253	− 0.131	0.307
HNRNPA2B1	–	–	− 0.413	0.005	–	–	–	–

### Verification of DERNAs in serum samples

**Fig. 8:**
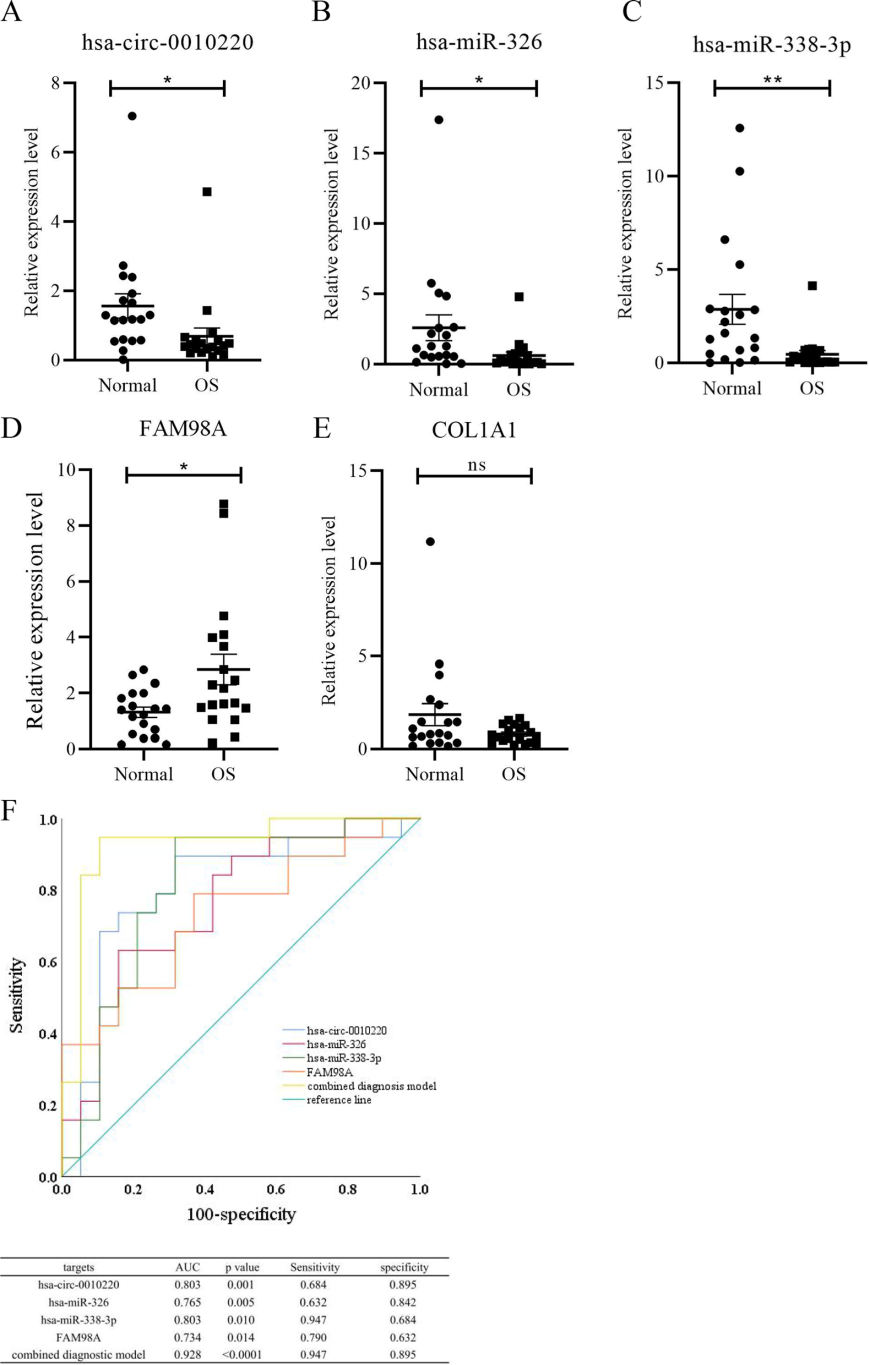
Validation the expression of DERNAs in serum sample by RT-qPCR. **A** Hsa-circ-0010220 was down-regulated significantly in OS serum sample compared with serum sample from healthy individuals (P < 0.05). **B** Hsa-miR-326 was down-regulated significantly in OS serum sample compared with serum sample from healthy individuals (P < 0.05). **C** Hsa-miR-338-3p was down-regulated significantly in OS serum sample compared with serum sample from healthy individuals (P < 0.01). **D** FAM98A was up-regulated significantly in OS serum sample compared with serum sample from healthy individuals. (P < 0.05) **E** COL1A1 was not significantly differential expression in OS serum sample compared with serum sample from healthy individuals (P = 0.09). **F** The ROC curve of hsa-circ-0010220, hsa-miR-326, hsa-miR-338-3p and FAM98A and the combined diagnostic model

The expression of FAM98A, COL1A1, their upstream targets, hsa-circ-0010220, hsa-miR-326, and hsa-miR-338-3p were validated by RT-qPCR of 19 OS serum samples and 19 serum samples from healthy individuals. Of these, hsa-circ-0010220 was found to be differentially expressed (Fig. 8A). The expression levels of hsa-miR-326 and hsa-miR-338-3p were significantly decreased in OS serum samples compared to serum samples from healthy individuals (Fig. 8B, C). Further, the expression of FAM98A was significantly increased in serum samples from OS patients (Fig. 8D). However, the results showed no difference in the serum expression level of COL1A1 in OS patients and healthy individuals (Fig. 8E). These data indicate that hsa-circ-0010220, hsa-miR-326, hsa-miR-338-3p, and FAM98A could be candidate biomarkers for early stage OS diagnosis and may be useful for prediction of the pathological progression of OS.

To further explore the diagnostic role of the OS validated targets, we performed ROC curve analysis and proposed a nice diagnostic model. Results demonstrated hsa-circ-0010220, hsa-miR-326, hsa-miR-338-3p, and FAM98A to have high diagnostic sensitivity and specificity. Their AUC values were more than 0.7. Of the four identified targets, hsa-circ-0010220 exhibited the highest specificity. As for sensitivity, hsa-miR-338-3p was the best biomarker. From this analysis, we evaluated a diagnostic model consisting of hsa-circ-0010220, hsa-miR-326, hsa-miR-338-3p, and FAM98A. The AUC value for the combined model reached 0.928, with specificity and sensitivity 89.47% and 94.74%, respectively (Fig. 8F).

## Discussion

CircRNA has long been considered to be non-functional and the product of abnormal gene splicing [26]. More recently, circRNA has been associated with the occurrence and development of various cancers [27–29]. OS is a common malignant orthopedic tumor. Although surgery, chemotherapy, and radiotherapy have significantly improved the survival rate of OS patients, patient prognosis is still very poor due to metastatic disease [3, 4]. Related studies have identified OS circRNAs as potential biomarkers [30, 31]. Herein, we identified key serum targets for OS diagnosis and metastatic prediction based on the final ceRNA network as well as immune cell infiltration patterns and characteristics. Further, immune cell infiltration patterns were shown to effectively distinguish OS patients from healthy individuals. By using serum targets and the model based on the final ceRNA network, diagnostic biological targets were identified, which performed better than those commonly used in clinical practice, ALP and LDH [5–7]. Most importantly, FAM98A was considered as a promising biomarker for OS metastasis.

Herein, OS raw datasets from the GEO public database were used to identify DEcircRNA, DEmiRNA, and DEmRNA expression profiles. On this basis, we conducted biological analysis of OS transcription factors, cellular functional characteristics, ceRNA and PPI networks in vivo. Further, PCA demonstrated the infiltration pattern of 22 types of immune cells to effectively distinguish OS patients from healthy controls. These results confirmed the value of the key serum biomarkers identified by the final ceRNA network based on immune cell infiltration. Further, pan-cancer analysis of large sample TCGA and GTEx databases, as well as analysis of world-wide clinical characteristics, verified the function of these targets and eliminated study errors. In comparison to other types of sarcoma, FAM98A and COL1A1 had good OS specificity. FAM98A was demonstrated to be diagnostic and predictive of metastasis within 5 years based on GSE21257, GSE124768, and GSE33382 datasets, which included 127 OS patients. We verified FAM98A, COL1A1, and their upstream targets in serum samples. Results showed FAM98A, hsa-circ-0010220, hsa-miR-326, and hsa-miR-338-3p to differentiate between OS serum samples and serum samples from healthy individuals as judged by qRT-PCR. ROC curve analysis established the specificity and sensitivity of the verified targets. The serum targets and the combined model based on them, are promising markers for OS diagnosis and metastatic prediction.

This study demonstrated the importance of M0 macrophages, M2 macrophages, and CD8+ T cells in OS. In a study by Zhang et al*.*, M0 macrophages and M2 macrophages were significantly related to prognosis [32]. Herein, the potential for identification of essential serum targets was verified using analysis of ceRNA network-related immune cell infiltration. Cell infiltration is an ideal biological target for cancer immunotherapy, especially with relationship to hsa-miR-326 and hsa-miR-338-3p. Each of which has the capacity to regulate M2 macrophages and cancer characteristics [33, 34]. Hsa-miR-326 is involved in the regulation of programmed death ligand 1 (PD-L1) and CD8+ T cells [35]. Transcription factors are important targets for non-coding RNAs. The top 5 differentially expressed transcription factors, EGR1, SP1, SP4, POU2F1, and NFIC, are all involved in immunotherapy [36–40]. Further, EGR1 is involved in the polarization of M2 macrophages induced by PD-L1, with anti-PD-L1 treatment promoting tumor resistance and metastasis through regulation of angiogenic osteoclastogenic factors [41, 42]. Based on this study, EGR1 should be explored as a valuable OS immunotherapy biomarker. Li et al*.* reported that POU2F1 induces cancer immune escape by increasing the expression of PD-L1 [43],. These relationships of OS are highlighted in this study. Bone is a highly specialized immune environment and the studies herein identify key serum biomarkers that provide for a better understanding of OS immunotherapy.

On the basis of GO and KEGG analysis, this study provides new perspectives. Allen et al*.* reported that in a zebrafish model of OS metastatic colonization, circulating tumors extravasate by down-regulation of immune pathways and the organization of the extracellular matrix [44]. In a study by Nie et al., deletion of STAG2 affected the cell cycle and promoted the expression of PD-1, enhancing immune system evasion by OS cells [45]. Mieszkowska et al*.* reported that collagen enriched in phenols significantly changed the phenotype of human OS cells, reducing immune inflammation [46]. Janus et al*.* reported that the NF-κB signaling pathway, which has an important role in both inflammation and the immune response, could be blocked by heat shock in OS without the involvement of the transcription factor, HSF1, or HSF1-induced heat shock proteins [47]. These attraction mechanism in OS pathology are further highlighted by our functional analysis. So, DEmRNAs identified by GO and KEGG analysis are worthy of further investigation with regard to cancer immunotherapy. In addition, the differential expression of hsa-circ-0010220 in serum also had a similar trend in OS tissues [48]. Regarding miRNA, Wang et al*.* reported that the long non-coding RNA, SNHG1, acting as a sponge for hsa-miR-326, regulated the expression of human NIN1 binding protein (NOB1), which affects the growth, migration and invasion of OS [49]. It is worth noting that results of this study have been verified in part by an independent investigation of OS associated hsa-miR-326. Cao et al*.* reported hsa-miR-326 to be an OS diagnostic and prognostic biomarker with reduced expression in advanced clinical stages and distant metastasis. Functionally, hsa-miR-326 was shown to regulate cell survival and apoptosis by targeting Bcl-2 in OS [50]. Little in vivo Bcl-2 functional work has been reported regarding OS metastasis and prognosis, making it difficult to elucidate the function of hsa-miR-326 in OS [51–53]. As such, the findings of this study deserve further attention with regard to the lower expression of hsa-miR-326 resulting in poorer clinical outcomes mediated by FAM98A. This study comprised patients of different racial backgrounds and from different geographic regions, and demonstrated that high levels of FAM98A expression are associated with metastasis and with metastasis five years after OS diagnosis. Most importantly, a new potential OS regulatory pathway was revealed that involved immune cell infiltration. Shao et al*.* reported that hsa-miR-326 regulates the expression of immune checkpoint molecules, PD-L1 and B7-H3, which affect the cytokine profile of CD8+ T cells as well as lung adenocarcinoma cell migration [35]. PD-L1 and B7-H3 have been identified as targets for immunotherapeutic intervention in OS [54]. The role of CD8+ T cells in OS is highlighted in this study. Further, hsa-miR-326 has been shown, through tumor-associated RohA, to play an important role in the immune response of M2 macrophages [33]. Furthermore, RohA has been reported to regulate the growth and metastasis of OS through ROCK and Wnt5a signaling pathways [55–57]. As such, the use of hsa-miR-326 as a new strategy for OS cancer immunotherapy may be reasonable.

Hsa-miR-338-3p was shown in this study to be involved in a novel regulatory axis. Previously, hsa-miR-338-3p was reported to be an OS tumor suppressor that targeted RUNX2 and CDK4 through the MAPK pathway [58]. Further, hsa-miR-338-3p has been shown to inhibit OS cell proliferation, migration, invasion, and epithelial–mesenchymal transition by targeting AHSA1 [59]. LncRNA CASC15 and circRNA CCDC66 serve as sponges for miR-338-3p, regulating OS progression [60, 61]. Herein we demonstrated differential serum expression of hsa-miR-338-3p, providing a simple detection method for OS diagnosis and prediction. Hsa-miR-338-3p is involved in immunological response mechanisms. For example, coagulation factor X, secreted in the TME, is a target of hsa-miR-338-3p. Coagulation factor X exhibits potent chemotactic activity, recruiting and promoting M2 macrophage polarization and regulation of tumor growth [34]. As such, hsa-miR-338-3p is a reasonable target for immunotherapy of OS. We have made a patent application for the herein described hsa-miR-338-3p primer. Hsa-miR-324-5p is involved in the novel regulatory axis constructed in this study. The axis regulates ACE1, which controls DNA repair and redox regulation [33], although its role in the pathogenesis of OS was not elucidated [62]. Molist et al. found that overexpression of hsa-miR-324-5p impaired cell proliferation and reduced tumor growth of rhabdomyosarcoma [63]. Therefore, further investigation of hsa-miR-324-5p function in OS is reasonable.

In this study, FAM98A was considered to be a novel biomarker for OS metastasis. Previously, FAM98A was shown to promote progression of endometrial carcinoma, non-small cell lung cancer, and breast cancer [64–66]. Moreover, FAM98A was related to poor prognosis and metastasis [64]. Most notably, FAM98A participates in the regulation of bone homeostasis, with inhibition resulting in bone resorption defects [67]. In this study, high levels of FAM98A expression were found in OS patients compared to normal subjects. Moreover, similar high expression were found in OS metastasis patients and patients with metastasis within five years of diagnosis compared to non-metastasis patients. As such, FAM98A is a promising non-invasive OS biomarker. No similar relationships were found for COL1A1. However, in hFOB1.19 cells heterozygous for the c.3781A allele and the c.3781C allele, the expression of COL1A1 was regulated by hsa-miR-345-5p, affecting ALP activity and substrate mineralization levels [68]. Hawkins et al*.* found that activation of Wnt/beta-catenin signaling affected the expression levels of COL1A1 in Ewing’s sarcoma [69]. It is worth noting that a prognostic value has been reported for COL1A1. For example, COL1A1 polymorphism rs1061970 has been associated with risk for OS and overall survival in Chinese patients [70]. Therefore, COL1A1 is a potential OS marker worthy of further study. RAN, another mRNA of the final ceRNA network, was identified in OS. RAN has been identified in various tumors, with high levels related to tumor aggressiveness and metastasis [71–73]. Jain et al*.* reported that hsa-miR-197-3p regulates fibrosarcoma carcinogenicity by targeting RAN [74]. It will be interesting to understand the unknown molecular mechanism behind the role of the RAN in OS. Moreover, HNRNPA2B1 has been reported to be an independent risk factor for OS. As an m6A-related regulator of the humoral immune response [75], it is an interesting biomarker for further study. In this study, OS had significant humoral immune involvement, with the final ceRNA network providing a novel understanding of HNRNPA2B1. HNRNPA2B1 may be an important regulator of OS.

Overall, this is the first report of the association of hsa-circ-0010220, hsa-miR-326, hsa-miR-338-3p, FAM98A with OS. Use of these molecules and the model based on network-related immune cell infiltration will be useful for OS diagnosis. Further, FAM98A is a promising biomarker for diagnosis and prediction of OS metastasis. Most importantly, the novel diagnostic model consisting of the four targets provided a 0.928 AUC value. Analysis of the significant immune characteristics of OS provides a novel perspective for further study of the identified targets. However, this study is limited by the small number of evaluated patients, which may have biased results. Validation of the results with a larger sample size is essential.

## Conclusions

Based on the analysis of OS immune cell infiltration characteristics, we identified, for the first time, hsa-circ-0010220, hsa-miR-326, hsa-miR-338-3p, and FAM98A as promising biomarkers for the diagnosis of OS. The combined assessment of these molecules provides for a novel diagnostic model. Most importantly, FAM98A was found to be useful for the diagnose and predict metastasis of OS. Within the context of the significant immune characteristics of OS described herein, we provided a novel perspective for future investigations of these potential OS biomarkers.

## Supplementary Information

**Additional file 1: Table S1.** RT-qPCR primers.

**Additional file 2: Figure S1.** A whole flow chart of this study.

## Data Availability

The datasets used and/or analyzed during the current study are available from the corresponding author on reasonable request.
