# Supplementary material for: Identification of key serum biomarkers for the diagnosis and metastatic prediction of osteosarcoma by analysis of immune cell infiltration
Source: Cancer Cell Int. 2022 Feb 12;22:78. doi: 10.1186/s12935-022-02500-6 (PMC8841093; doi:10.1186/s12935-022-02500-6)
Supplement: Supplementary file 1 — Additional file 1: Table S1. RT-qPCR primers. [file 12935_2022_2500_MOESM1_ESM.pdf]

Table S1 RT-qPCR primers

Target Name	Primer Type	Target Sequence
hsa-circ-0010220	Forward	AGCTCTGGGAGATGCTGGAA
	Reverse	TATGGCAGGCTGTGGAGGAG
FAM98A	Forward	TTGGAGTCGTTGGAAGATCTAG
	Reverse	AGCACTCTTAATTCAGACACCA
COL1A1	Forward	AAAGATGGACTCAACGGTCTC
	Reverse	CATCGTGAGCCTTCTCTTGAG
hsa-miR-326		TATAATCCTCTGGGCCCTTCCTCC
hsa-miR-338-3p		CGCAACAATATCCTGGTGCTGAGTG
GAPDH	Forward	CAGGAGGCATTGCTGATGAT
	Reverse	GAAGGCTGGGGCTCATTT
U6	Forward	TCGCTTCGGCAGCACA
	Reverse	AACGCTTCACGAATTTGCGT
