# Supplementary figures and images for: Identification of key serum biomarkers for the diagnosis and metastatic prediction of osteosarcoma by analysis of immune cell infiltration
Source: Cancer Cell Int. 2022 Feb 12;22:78. doi: 10.1186/s12935-022-02500-6 (PMC8841093; doi:10.1186/s12935-022-02500-6)

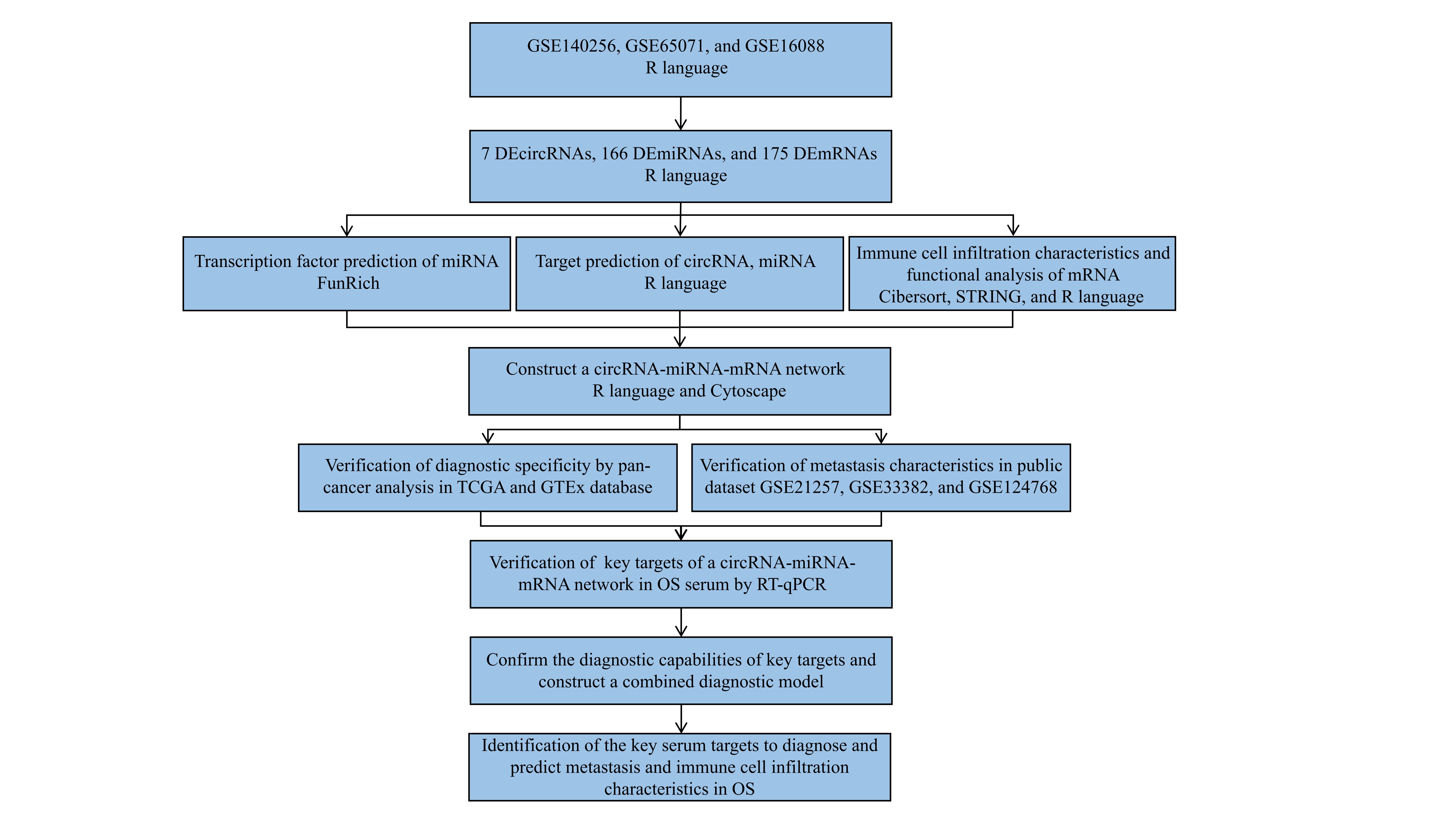

Supplement: Supplementary file 2 — Additional file 2: Figure S1. A whole flow chart of this study. [file 12935_2022_2500_MOESM2_ESM.jpg]
